# Evaluation of dimensional behavior of peri-implant tissues in implants immediately exposed or submerged in fresh extraction and healed sites: a histological study in dogs

**DOI:** 10.1186/s40729-018-0120-z

**Published:** 2018-02-12

**Authors:** Sergio Alexandre Gehrke, Leana Kathleen Bragança, Eugenio Velasco-Ortega, José Luis Calvo-Guirado

**Affiliations:** 1 Biotecnos Research Center, Calle Cuareim, 1483, CP: 11.100, Montevideo, Uruguay; 2 University Catholica San Antonio de Murcia (UCAM), Murcia, Spain; 3 Implant Dentistry, Seville University, Seville, Spain; 4 General Dentistry, Seville University, Seville, Spain; 5 Implant Dentistry Master, Seville University, Seville, Spain; 6 International Dentistry Research Cathedra, Faculty of Medicine and Dentistry, San Antonio Catholic University of Murcia (UCAM), Murcia, Spain

**Keywords:** Crestal bone behavior, Fresh extraction sites, Exposed implants, Submerged implants

## Abstract

**Background:**

The aim of this study was to compare histologically the dimensional behavior of peri-implant tissues during osseointegration of immediately exposed or submerged implant placement in fresh extraction and healed sites.

**Methods:**

Four fresh extraction and four delayed implant sites were placed in each hemimandible of five dogs at the bone crest level. In 2 implants of each side were installed a healing abutment (exposed) and two cover screw (submerged) and formed four groups: implant installed in fresh extraction submerged (group 1), implants in fresh extraction immediately exposed (group 2), implants installed in healed site submerged (group 3), and implants in healed site immediately exposed (group 4). After 12 weeks of healing period, histomorphometric analyses of the specimens were carried out to measure the crestal bone level values and the tissue thickness in the implant shoulder portion.

**Results:**

The measure of crestal bone level showed some higher values for implants installed in fresh extraction sites in the buccal aspect: 1.88 ± 0.42 mm for group 1 and 2.33 ± 0.33 mm for group 2, with statistical significance among all four groups tested (*P* < 0.001). For peri-implant tissue thickness, a significative higher statistical difference (*P* < 0.001) for implants installed in healed sites (groups 3 and 4) was found.

**Conclusions:**

Within the limitations of the present animal study, our findings suggest that the implants placed in fresh extraction or healed site and with regards to the moment of exposition (immediately or no) are important factors to the amount of peri-implant tissues after remodeling over a period of 12 weeks. The null hypothesis was rejected.

## Background

After the tooth loss, there is a progressive involution of the alveolar bone both in the horizontal and the vertical dimensions [1, 2]. Moreover, the most rapid reduction in the alveolar bone after tooth extraction occurs during the first 3 months [3, 4]. Implants immediately positioned in alveolus after the surgical extraction of the tooth exhibit a success ranging from 92.7 to 98.0% [5]. Some authors suggested that immediate implant placement may counteract the bone remodeling process and preserve the dimension of the alveolar ridge [6–8]. However, multiple animal investigations have failed to support this hypothesis [3, 9]. In this sense, studies by Araújo et al. [3, 10] found a pronounced resorption of the buccal and lingual bony walls after immediate placement in fresh extraction sockets. In long-term observations, no significant differences in the success and esthetic outcomes have been reported between immediate and delayed implants [11–13].

The surgical requirements for ideal immediate implants in fresh alveolus include atraumatic tooth extraction, preservation of the extraction socket walls, and thorough alveolar curettage to eliminate any possible pathological material [14, 15]. Also, primary implant stability is also an essential requirement and is achieved through the use of implants that exceed the alveolar apex by 3–5 mm or by placing a dental implant with a greater diameter than the alveolar socket [16, 17]. Gehrke et al. [18] demonstrate that the stabilities of the implants placed into fresh extraction sockets or at healed alveolar sites exhibited similar ISQ value evolutions across the three investigated time points (0, 90, and 150 days).

Non-submerged implants showed comparable clinical results to submerged implants and resulted in higher patient satisfaction due to decreased surgical intervention [19]. In this regard, Abrahamsson et al. [20] compared the mucosa and the bone tissue surrounding implants non-submerged or submerged and observed that parameters such as the length of the barrier epithelium of the peri-implant mucosa, the height of the zone of connective tissue integration, the level of the marginal bone, and the density of bone between threads were almost identical in the two experimental groups at the end of the healing period.

Then, the good results were obtained with both techniques (implants placed into fresh alveolus and implants non-submerged); these have been joined together with the objective to reduce the time of the treatment. However, the esthetic results can directly influence by the peri-implant tissue dimension (vertical or horizontal) and position in relationship of the cervical implant portion. In this way, the objective of the present study was to compare the dimensional changes of crestal bone level and peri-implant soft tissue during osseointegration of immediately exposed and submerged implants placement in fresh extraction or healed sites using a mandible dog model. The null hypothesis was the moment of implant placement after tooth extraction (immediate or after healing) or leaving the implant exposed or submerged not affecting the behavior of the peri-implant tissues.

## Methods

### Implants and abutments

**Fig. 1:**
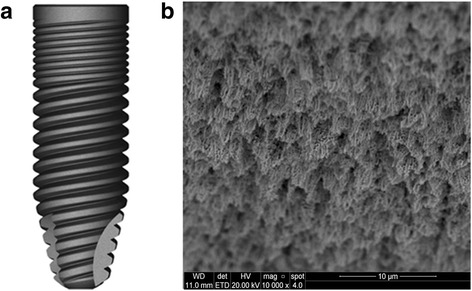
Image of the implant (**a**) and surface (**b**) used in the present study

A total of 40 implants were installed (ICI implant, Galimplant, Sarria, Spain), with 3.5 mm in diameter by 10 mm in length. Eight implants in each dog, half per hemimandible. The surface treatment of this implant model is developed by blasting with three different granulometries of Al_2_O_3_ and pickling using a hydrofluoric solution (HF) at low temperature and short time, which aims to remove any traces of Al_2_O_3_. Plus, the conditioning of the surface was performed using hydrochloric acid solution (HCl) and sulfuric acid (H_2_SO_4_) at high temperature and short time (Fig. 1). Twenty titanium healing abutments with 3.5 mm in diameter and 6 mm in length were used.

### Surgical procedure and animals care

Five American foxhound dogs of approximately 1 year of age were used in this study. The Ethics Committee for Animal Research at The University of Murcia (Spain) approved the study protocol, which followed the guidelines established by the European Union Council Directive of February 2013 (R.D.53/2013). Clinical examination determined that all animals were in good general health; moreover, all animals presented intact maxillae, without occlusal trauma or mucosal lesions.

The animals were pre-anesthetized with acepromazine 0.12–0.25 mg/kg, buprenorphine 0.01 mg/kg, and medetomidine 35 mg/kg. This mixture was injected intramuscularly in the femoral quadriceps. Animals were then taken to the operating theater, where an intravenous catheter was inserted into the cephalic vein, and propofol (0.4 mg/kg/ min) was continuously infused to maintain the general anesthesia. Conventional dental infiltration anesthesia (articaine 40 mg, 1% epinephrine) was injected at the surgical intraoral sites. All procedures were carried out under the supervision of a veterinary surgeon.

Initially, an impression of each hemimandible was performed to make a surgical guide indicate the implant position, which was predetermined to correspond with the distal root and the center of the crown teeth. Sixty days previous to the surgery, the left mandibular premolars (P2, P3, P4) and molar (M1) were extracted to heal the alveolus sites [21]. In the surgery to place the implants, equally to previous surgery, the teeth of the right hemimandibles were sectioned in a bucco-lingual direction using a tungsten carbide bur so that the roots could be extracted individually without damaging the remaining bony walls. After that, full-thickness mucoperiosteal flaps were increased. The socket of the distal root of each premolar was used as experimental site. For the left sides, a full-thickness mucoperiostal flap was used. All implants were positioned in the crestal bone level. After implant placement, a randomization (randomization.com) was performed to determine which implants received healing abutment and the submerged implants, forming four groups: implant installed in fresh extraction and submerged (group 1), implants in fresh extraction and immediately exposed (group 2), implants installed in healed site and submerged (group 3), and implants in healed site and immediately exposed (group 4). The height of the healing abutments was determined to stay 0.5 mm less of the contact with the corresponding antagonist teeth. No grafting materials were used between the implants and the bony plates. The flaps were closed using single nonabsorbable sutures (Silk® 4-0, Sweden & Martina, Due Carrare). After the surgical procedures, animals received antibiotic treatment (amoxicillin 500 mg, twice a day) and analgesics (ibuprofen 600 mg, three times a day) via the systemic route. Moreover, dogs were fed a soft diet for 7 days, and plaque control was maintained by the application of Sea 4 (Sea 4 teeth, Blue Sea Laboratories, Alicante, Spain). Wounds were inspected daily for clinical postsurgical complications. Two weeks after surgery, sutures were removed. All animals were sacrificed at 12 weeks after the implant insertion by means of an overdose of Pentothal Natrium® (Abbott Laboratories, Madrid, Spain).

### Histological preparation and histomorphometric analysis

**Fig. 2:**
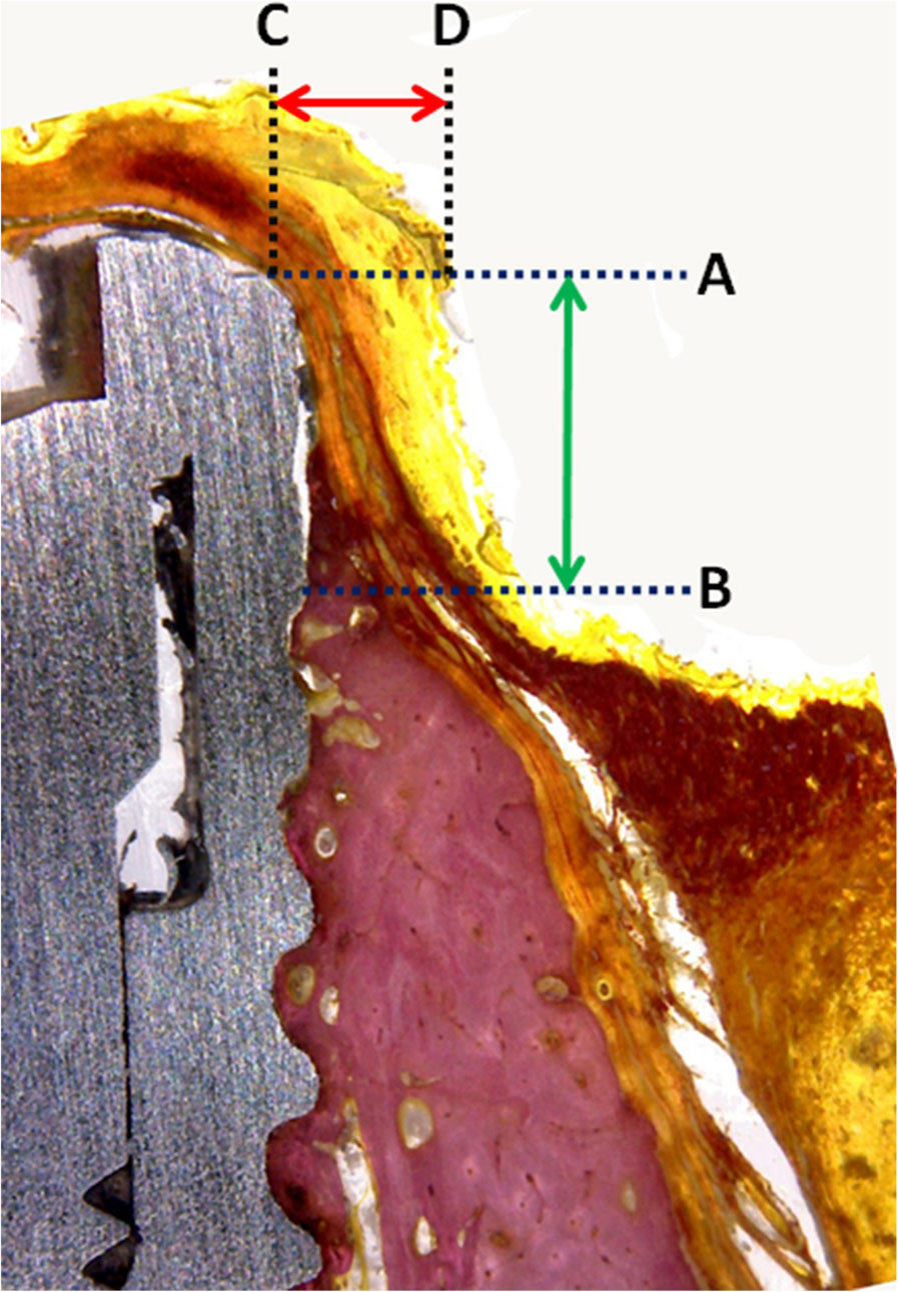
Parameters measured in each group. Crestal bone loss is the distance between the implant collar (A) and the first bone contact of the crestal bone (B) = A-B bone height; and, the tissue thickness that is the distance from the implant collar (C) to the more external portion of the tissues (D) = C-D tissue thickness. Picrosirius red staining. Original magnification × 16

The hemimandibles were removed with care to preserve the integrity of both peri-implant hard and soft tissues, washed in saline solution and fixed in 10% buffered formalin, and sent for processing at the Laboratory of Ucam-Biotecnos (Murcia, Spain). Specimens were dehydrated in ascending series of alcohol rinses and embedded in a glycol methacrylate resin (Technovit 7200 VLC; Kulzer, Wehrheim, Germany). After polymerization, the specimens were sectioned along its longitudinal axis with a high-precision diamond disk in the IsoMet® 1000 (Buehler, Lake Bluff, IL, USA), at about 150 μm down to 30 μm. A total of two slides were obtained for each implant. The slides were stained with Picrosirius Red Stain (Polysciences, Inc., Warrington, USA) and observed in a normal transmitted light microscope and a polarized light microscope (Nikon, Tokyo, Japan). Buccal bone wall level in comparison with lingual bone wall height after remodeling was expressed as a linear measurement from the implant shoulder to the first bone-implant contact, showed in the Fig. 2 corresponding with the A-B distance. The buccal and lingual tissue thickness was measured in the level corresponding with the implant shoulder (A line) from the implant to the external epithelium portion of the mucosa, showed in the Fig. 2 corresponding with the C-D distance. The measurements were performed by an expert examiner in histology (SG).

Metric evaluation of the predetermined parameters was carried out using a light microscope (Nikon, Tokyo, Japan) connected to a high-resolution video camera (3CCD, JVC KY-F55B, JVC®; JVC, Yokohama, Japan). After digitizing the phase of each specimen under the light microscope, all proposed details were measured in the images using the program Image Tool version 5.02 for *Microsoft Windows*™ (UT Health Science Center School of Dentistry, San Antonio, TX, USA).

### Statistical analysis

Means, medians, and standard deviations of crestal bone height and tissue thickness were calculated for all groups. All data sets (*n* = 10) were tested for normality using the Shapiro–Wilk test, and the data did not show normal distribution. The Friedman test was performed for intergroup comparisons in buccal or lingual recorded measures for A-B and C-D parameters followed by the Dunn’s multiple comparison test for further comparison of different groups. Furthermore, Wilcoxon matched-pairs signed-rank test was used for the comparison of two groups. The significance level was set at *P* < 0.05.

A power analysis was conducted to determine appropriate sample size; although it was determined that 6 samples from each group would generate a 95% confidence limit (G3Power), 10 samples were proposed for each situation to increase the level of significance.

## Results

The surgical sites healed uneventfully. All animals presented appropriate healing during the first week following the surgical procedure. Post-surgical inspections for 2 weeks post-operatively indicated the absence of infection or inflammation. All implants presented osseointegration after the proposed period and were available for histological analysis.

### Histological observations

**Fig. 3:**
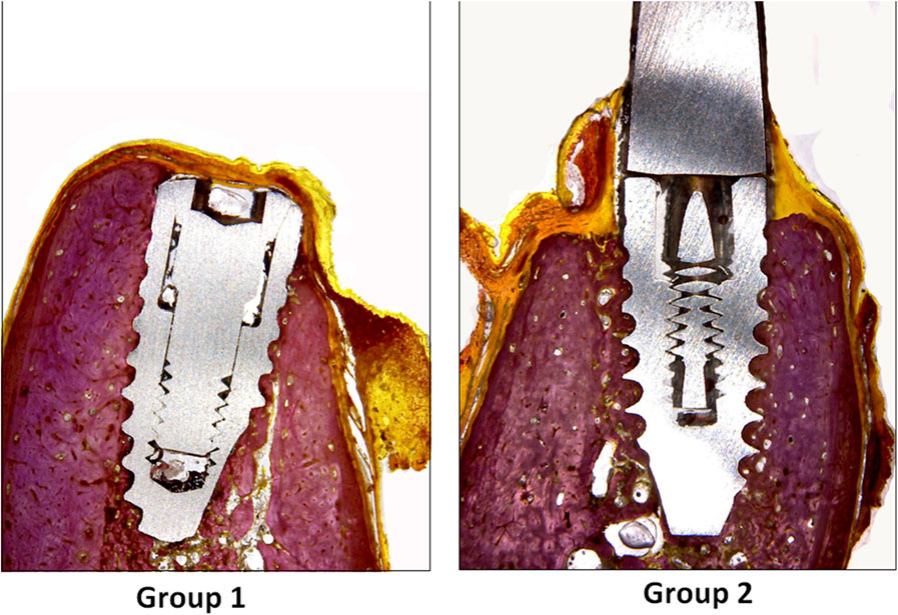
Images of groups 1 and 2 representing the implants place in fresh sockets sites. Picrosirius red staining. Original magnification × 4

**Fig. 4:**
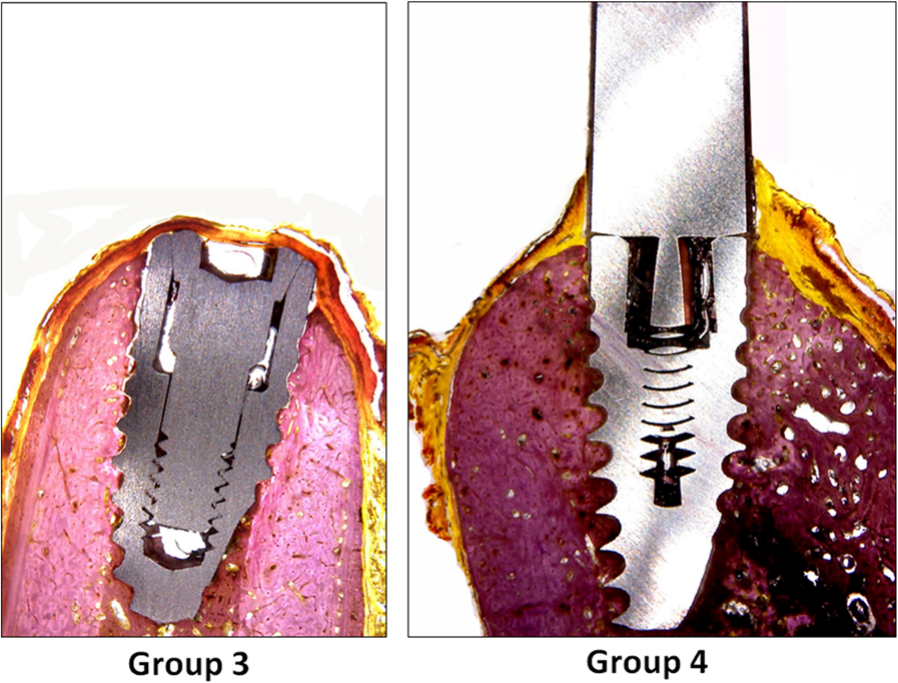
Images of groups 3 and 4 representing the implants place in healed alveolar sites. Picrosirius red staining. Original magnification × 4

Direct contact was observed between living bone, and all implants without the presence of soft tissues were observed in all groups. However, during the healing, the crestal areas were accompanied by decreases in the dimensions of the buccal as well as the lingual bone walls in different proportions for each group (Figs. 3 and 4). For all implants, keratinized oral epithelium was continuous with junctional epithelium facing the implants and the healing abutments. Subjacent connective tissue with a dense network of collagen fibers was observed.

### Bone-to-shoulder height measurements

**Fig. 5:**
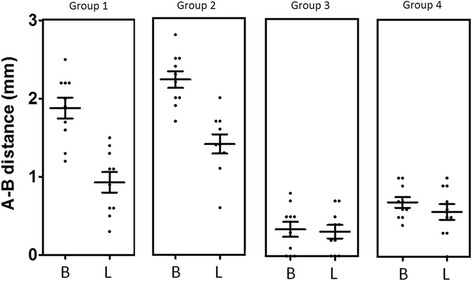
Graph comparing the data of buccal (B) and lingual (L) measured the A-B distance (bone height). Group 1 = implant installed in fresh extraction and submerged; group 2 = implants in fresh extraction and immediately exposed; group 3 = implants installed in healed site and submerged; and group 4 = implants in healed site and immediately exposed

After evaluating all measurements, the distance from the top of the implant collar (line A) to the first contact of the implant with the bone (line B) was measured for buccal and lingual aspect. Mean, median, standard deviation, and standard error for each group evaluated for lingual as well as buccal sites are summarized in Table 1 and showed in the Fig. 5. The buccal and lingual dimensions showed statistically significant differences at 12 weeks among the groups, which are showed in the Table 3 and the distribution data represented in the Fig. 7a. A-B distance evaluated in the group 3 was significantly lower in both buccal and lingual measured groups, whereas for group 2, A-B distance resulted higher than the other groups.

**Table 1:** Mean, median, standard deviation, and standard error for each group evaluated for lingual as well as buccal sites of the crestal bone height (in mm) for all groups

	Crestal bone loss (A-B distance in mm)							
	Buccal				Lingual			
	Group 1	Group 2	Group 3	Group 4	Group 1	Group 2	Group 3	Group 4
Median	1.95	2.25	0.40	0.65	1.00	1.40	0.30	0.55
Mean	1.88	2.23	0.34	0.69	0.93	1.41	0.31	0.57
Std. deviation	0.42	0.33	0.30	0.21	0.41	0.38	0.27	0.32
Std. error	0.13	0.11	0.10	0.07	0.13	0.12	0.09	0.10
Lower 95% CI of mean	1.58	1.99	0.12	0.54	0.63	1.14	0.12	0.34
Upper 95% CI of mean	2.18	2.47	0.56	0.84	1.23	1.69	0.51	0.80

### Buccal and lingual tissue thickness measurements

**Fig. 6:**
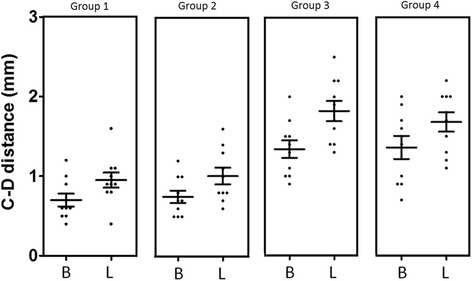
Graph comparing the data of buccal (B) and lingual (L) measured the C-D distance (tissue thickness). Group 1 = implant installed in fresh extraction and submerged; group 2 = implants in fresh extraction and immediately exposed; group 3 = implants installed in healed site and submerged; and group 4 = implants in healed site and immediately exposed

**Fig. 7:**
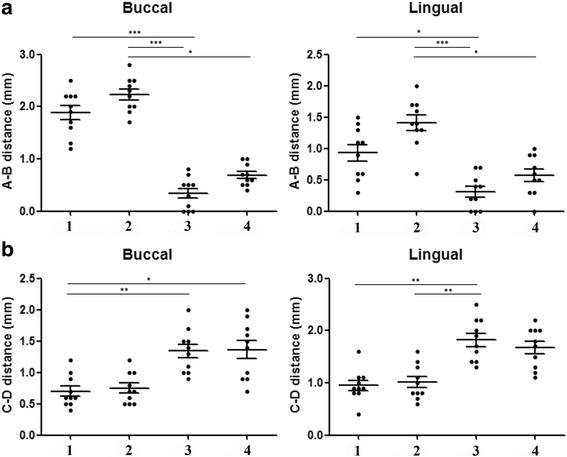
Multiple graphs comparing A-B distance (height bone) (**a**) and the C-D distance (tissue thickness) (**b**) among different groups. Differences between groups were assessed by Dunn’s multiple comparison test (*P < 0.05; **P < 0.01; ***P < 0.001). 1 = (group 1) implant installed in fresh extraction and submerged; 2 = (group 2) implants in fresh extraction and immediately exposed; 3 = (group 3) implants installed in healed site and submerged; and 4 = (group 4) implants in healed site and immediately exposed

The overall mean of the tissue thickness from the top of the implant collar (line C) to the more external portion of the tissues (line D) was also calculated. Parameters such as mean, median, standard deviation, and standard error for each group evaluated for lingual as well as buccal sites are summarized in Table 2 and showed in the Fig. 6. Crestal bone height was higher for group 2 both at buccal and lingual sites. The statistical analysis also revealed differences among groups of the measured parameters, which are presented in the Table 3 and the distribution data represented in the Fig. 7b.

**Table 2:** Mean, median, standard deviation, and standard error for each group evaluated for lingual as well as buccal sites of the tissue thickness (in mm) for all groups

	Tissue thickness (C-D distance in mm)							
	Buccal				Lingual			
	Group 1	Group 2	Group 3	Group 4	Group 1	Group 2	Group 3	Group 4
Median	0.60	0.70	1.35	1.45	0.90	0.90	1.80	1.75
Mean	0.70	0.75	1.34	1.36	0.95	1.01	1.82	1.68
Std. deviation	0.25	0.25	0.35	0.46	0.30	0.33	0.41	0.39
Std. error	0.08	0.08	0.11	0.14	0.10	0.10	0.13	0.12
Lower 95% CI of mean	0.52	0.57	1.09	1.03	0.73	0.77	1.53	1.40
Upper 95% CI of mean	0.88	0.93	1.59	1.69	1.17	1.25	2.11	1.96

**Table 3:** Statistical analysis comparing measured distances (A-B and C-D) among different groups in buccal and lingual sites

		Group					
		1	2	3	4	Friedman test	Friedman statistic
		Mean ± SD	Mean ± SD	Mean ± SD	Mean ± SD		
Crestal bone loss (A-B distance)	Buccal	1.88 ± 0.42^a^	2.23 ± 0.33^bc^	0.34 ± 0.30^ab^	0.69 ± 0.21^c^	*P* < 0.0001	26.45
	Lingual	0.93 ± 0.41^d^	1.41 ± 0.38^ef^	031 ± 0.27^de^	0.57 ± 0.32^f^	*P* < 0.0001	21.43
Tissue thickness (C-D distance)	Buccal	0.70 ± 0,.25^gh^	0.75 ± 0.25	1.34 ± 0.35^g^	1.36 ± 0.46^h^	*P* = 0.0005	17.79
	Lingual	0.95 ± 0.30^i^	1.01 ± 0.33^j^	1.82 ± 0.41^ij^	1.68 ± 0.39	*P* = 0.0003	18.68

Different superscript letters in the same row indicate significant differences between groups assessed by Dunn’s multiple comparison test (*P <* 0.05) and Wilcoxon signed rank test for comparison of different groups

^a^*P =* 0.0059

^b^*P =* 0.0055

^c^*P =* 0.0053

^d^*P* = 0.0125

^e^*P* = 0.0056

^f^*P* = 0.0058

^g^*P =* 0.0039

^h^*P =* 0.0080

^i^*P =* 0.0058

^j^*P =* 0.0039

## Discussion

The immediate implants in fresh sockets have demonstrated a great success rate [18, 22–24]. However, the removal of a single tooth followed by immediate placement of an implant results in marked alterations of the ridge in the horizontal as well as in the vertical dimension. The early phases of tissue integration in immediate post-extraction implants have been well documented [1, 25, 26]. The implants non-submerged are used with the intention of reducing the treatment time and decrease the quantity of surgical interventions, fleeing the protocol initially proposed by Branemark. Also, several studies have demonstrated a high rate of success when compared with the traditional technique [27–29]. However, it is a consensus that the implantation technique in alveolus immediately after tooth extraction and the use of immediate load is predictable in terms of osseointegration, standing as the main point of the behavior of the peri-implant tissue around of these implants. In this sense, the present investigation showed the tissues’ behavior after 12-week healing period which affected both buccal and lingual crestal bone and the tissue thickness in the portion corresponding to the implant collar and so the null hypothesis was rejected.

The conservation of bone around the implant especially in the buccal plate plays a crucial role on esthetics. Resorption of buccal plate may lead to exposed threads thus affecting the esthetic of the treatment, even if prostheses are not still connected [30]. In this sense, Calvo-Guirado et al. [31] showed that the resorption of the buccal plate was more pronounced in implants installed in fresh sockets, corroborated by the results of the present study, which revealed greater depth of crestal bone resorption at the buccal crest than at the lingual crest. Moreover, this bone dehiscence following implant placement corroborates findings reported previously [2, 3, 31–33]. In the present study, the crestal buccal and lingual bone height after the remodelation decreased in the implants without immediate load and, mainly, in implants placed in healed alveolar sites from the 12-week healing period. The study of Araújo et al. [10] corroborated this fact; the authors concluded that the implant placement failed to preserve the hard tissue dimension of the ridge following tooth extraction, both in the buccal and the lingual bone walls that were resorbed.

In the present study, the implants were positioned in the crestal bone level, by following Bornstein et al. [34, 35] which reported that the implants are often inserted within the bone crest. Tomasi et al. [36] in a clinical trial observed that the implant position conditioned the amount of buccal crest resorption. Moreover, the thickness of the buccal bone plate and the tridimensional positioning of the implant must be considered because these are important factors that influence the response of hard tissues during healing. In this sense, each animal was performed a surgical guide, based in the previous natural teeth, to position the implants in all groups and conditions in the same place because mainly in the site with the presence of alveolus post-extraction, this condition induces the error of the ideal position during the implant osteotomy.

In relation to the non-submerged implants, it has become a widely reported practice with success rates ranging from 82.9 to 95.7% [37–39]. Theoretically, submerged implant during the osseointegration period are less susceptible to complications; however, some studies comparing submerged implants and non-submerged showed no difference in the implant failure rate, postoperative infection, and marginal bone loss [40]. In the present study, the two groups with non-submerged implants compared between them (groups 1 vs 2 and, groups 3 vs 4), the bone height was smaller, which is likely related to the presence of micromovements generated during mastication during the initial period of osseointegration [41].

Today, implants with expanded platform have demonstrated better crestal bone preservation. Then, in this study, it was carried out by the insertion of implants with an expanded platform and a surface characterized for presenting light roughness in the upper part of the neck, different parts of the body, and apical portion where showed a highly roughness. Previous studies had established that the use of implants with a rough surface may influence the amount of bone regeneration and the values of BIC during healing [9, 20, 42]. Different studies have assessed that implants presenting a rough surface may influence the degree of bone regeneration and the percentages of BIC during healing [9, 43, 44]. Calvo-Guirado et al. [31, 45] concluded that the surface treatment can reduce the crestal bone resorption. Cooper [46] found that an increased surface roughness improves bone integration of the implant, increases osteoconduction, and increases osteogenesis.

New studies are needed to define the influence of other surface compositions and neck configurations for implants placed in fresh extraction sockets with/or without submerged and the influence of abutment change on crestal bone stabilization during the remodeling process. These would appear to be important factors for improving peri-implant bone and soft tissue stability and clinical outcomes, including esthetics, which are of particular importance in the anterior zone.

## Conclusions

Within the limitations of this study, our findings suggest that the crestal bone height is larger when implants are inserted in healed areas in comparison with implants installed in fresh extraction sites. Moreover, significant differences were found between non-exposed and immediately exposed implants with regards to crestal bone height position, and higher thickness tissue values in the groups of healed sites implants were found.
